# Beyond the womb: pontine hemorrhage’s impact on maternal and fetal outcomes: a case report from Nepal

**DOI:** 10.1097/MS9.0000000000002455

**Published:** 2024-08-07

**Authors:** Jeshika Yadav, Kiran Bhusal, Ishwor Thapaliya, Khagendra Bhandari, Swati S. Jha, Ramesh Khadayat, Asmita Ghimire

**Affiliations:** Tribhuvan University, Institute of Medicine, Maharajgunj, Nepal

**Keywords:** pontine hemorrhage, pregnancy, stroke

## Abstract

**Introduction:**

Strokes cause 37% of cardiovascular-related deaths in women, with 40% during delivery and 50% postpartum. Pontine hemorrhage, a rare cerebrovascular disease, can occur during pregnancy and postpartum, requiring complex diagnosis and management.

**Case presentation:**

A 33-year-old primipara female presented with headache, vomiting, and altered consciousness at 15 weeks and 5 days gestation. She had no history of head trauma, involuntary urine passage, cough, shortness of breath, palpitation, or per vaginal bleeding. Psychiatric and neurology consultations were conducted to address her symptoms.

**Discussion:**

Pontine hemorrhage is a rare condition affecting pregnant women, causing mild symptoms like vertigo, ataxia, and dysphagia. Our patient underwent an initial blood investigation with imaging of her brain (CT and MRI) to establish the diagnosis of pontine hemorrhage. It is believed to be caused by hormonal fluctuations, but studies have not proven it. Stroke during pregnancy is rare, with only 10% occurring during the antepartum period. Diagnosis requires a multidisciplinary approach with history, clinical examination, and radiological imaging like CT and MRI. Treatment involved antiplatelet therapy, which ensured favorable maternal and fetal outcomes for our patient.

**Conclusion:**

In summary, this case demonstrates the rare incidence of pontine hemorrhage during pregnancy. It highlights the diagnostic challenges and successful treatment with antiplatelet therapy, emphasizing the necessity for a comprehensive approach to ensure favorable maternal and fetal outcomes.

## Introduction

Highlights

- Cerebral pontine infarctions are uncommon but should be considered in pregnant and non-pregnant women presenting with CNS symptoms indicative of a cerebral vascular accident.
- Diagnosing hemorrhagic strokes in pregnant women involves a holistic approach like clinical history, physical examination, and low fetal exposure radiographic imaging, such as CT and MRI.
- Acute pontine hemorrhage in pregnancy demands a collaborative approach among obstetric, neurological, and surgical specialists for optimal management and outcome.
- Delaying delivery is an option with reassuring fetal monitoring and ongoing maternal resuscitation, favoring vaginal delivery unless obstetrical indications suggest otherwise.

Strokes account for 37% of cardiovascular-related deaths in women globally, with around 40% occurring during delivery, 50% in the postpartum period, and 10% in the antepartum period^1^. Pontine hemorrhage is a rare type of intracerebral hemorrhage (ICH) that can occur during pregnancy and the postpartum period resulting in significant morbidity and mortality for both the mother and the fetus. The incidence of strokes in pregnant patients is estimated at 34.2 per 100 000 deliveries, and the mortality rate is 1.4 per 100 000^2^, deliveries, accounting for both hemorrhagic and ischemic types of strokes. Pregnancy-related pathophysiological changes increase the likelihood of cerebrovascular disease in pregnant women^1^. The risk of ICH remains at its highest level during the third trimester of pregnancy and the initial 12 weeks following childbirth^3^. Diagnosing and managing ICH during pregnancy is complex due to dual risks for both mother and fetus^4^. Pontine strokes are uncommon compared to other ischemic events that can lead to substantial disability and nerve palsies. Thus, medical providers should consider these conditions when female patients show CNS symptoms suggestive of a cerebral vascular accident^1^. Imaging tests like computed tomography (CT) and magnetic resonance imaging (MRI) can be used in the diagnosis of pregnancy-related ICH. However, MRI is preferred for evaluating brainstem stroke because lesions of pontine stroke may be missed in CT^1,4^.

Here, we report a case of a pregnant woman presenting with headache, vomiting, and altered consciousness who was diagnosed with acute infarcts in the right cerebellar hemisphere, pons, and right cerebral peduncle on MRI, which is by the CARE checklist^5^.

## Case presentation

A 33-year-old primipara female presented to the emergency department at 15 weeks and 5 days of gestation [calculated from the last menstrual period (LMP)] with chief complaints of headache, vomiting, and altered consciousness for 1 day. The headache was acute in onset, non-radiating in the bilateral frontotemporal region, and associated with dizziness and blurred vision. She had two episodes of non-projectile vomiting containing food particles without any bile or blood staining. She also had altered consciousness, reduced responsiveness, inability to recognize family, and impaired vocalization with incomprehensible speech followed by sudden weakness in the left upper limb progressing to the left lower limbs. She had no history of head trauma or involuntary passage of urine. She had no cough, shortness of breath, palpitation, or per vaginal bleeding. There is a history of the previous lower segment cesarian section without insignificant events. There is no significant history of any past medical illnesses.

She consumes a mixed diet and belongs to a middle-class family, according to Modified Kuppuswamy’s socioeconomic scale^6^.

At the presentation to the hospital, she was ill-looking with a pulse rate of 102 beats/min, regularly regular with a blood pressure of 130/70 mmHg. Her respiratory rate was 22 breaths /min with SpO_2_ of 96% and temperature of 97.2°F. Her BMI was 18.2 kg/m^2^. She had a Glasgow Coma Scale (GCS) score of 10/15 (i.e. eye response: 3, motor response: 5, verbal response: 2). She was not oriented to time, place, or person with impaired speech and sluggish left pupil. There was no facial palsy. On motor examination, there was decreased power with increased tone and exaggerated deep tendon reflexes in the left upper and lower limbs. Babinski’s reflex was upgoing bilaterally. There were no other remarkable systemic findings. Based on the clinical history and physical examination, viral meningoencephalitis and right-side cerebrovascular accidents were considered differential diagnoses.

Initial laboratory investigations revealed increased total leukocyte count (13 500/cm, normal range: 4000–11 000/cm), neutrophilia (81%; normal range: 45–75), lymphocytosis (13%; normal range: 25–45), low hemoglobin concentration (11.5 gm%; normal range: 12–18), decreased packed cell volume (32.7%, normal range: 36–54), decreased RBC count (3.89 million/mm^3^), hyponatremia (128 mEq/l; normal range: 134–146), hypokalemia (3.5 mEq/l; normal range: 3.6–5.0), and low serum creatinine (0.4 mg/dl; normal range: 0.5–1.2). The serological test for dengue fever (antibody IgG, IgH, and NS1 antigen) was negative. The anticardiolipin antibodies IgG (0.69 PL-IgG-U/ml) and IgM (2.22 PL-IgM-U/ml) were within the normal range (<12 U/ml) along with β-2 glycoprotein 1 IgG (2.117 U/ml; normal range: <20 U/ml) but raised fibrinogen (533 mg/dl; normal range: 200–400).

**Figure 1:**
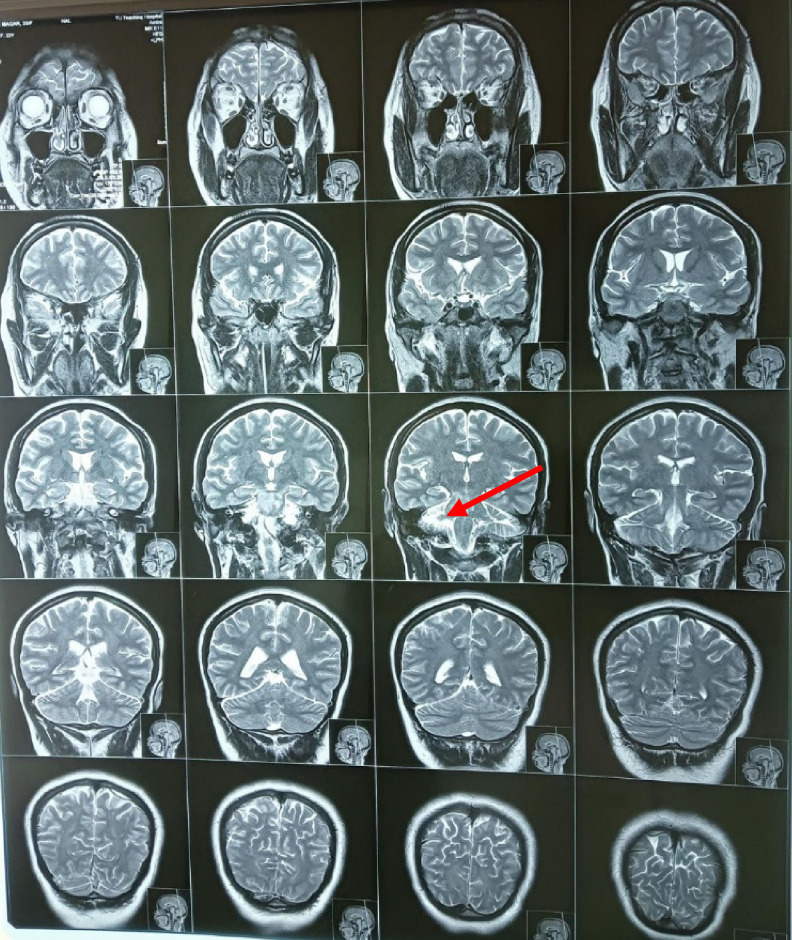
MRI study: There is a focal area of T2/FLAIR high signal intensity noted in the right cerebellar hemisphere.

**Figure 2:**
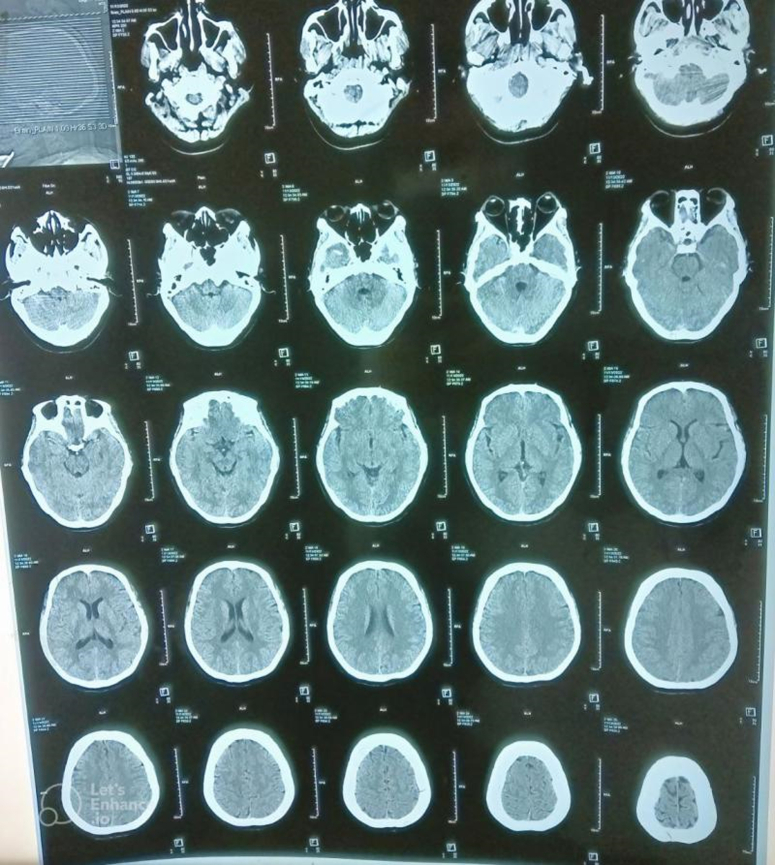
A non-contrast CT of the head.

As shown in Figure 1, the MRI scan of the brain showed an acute infarct in the right pons and right cerebellar peduncle. The occlusion of the basilar artery with obstruction of the V3 segment right vertebral artery was shown by the MRA study with normal MRV study. A non-contrast CT scan of the brain showed no acute intracranial pathology, as shown in Figure 2. The electrocardiogram showed mild tricuspid regurgitation, with 60% left ventricular ejection fraction, and was negative for intracardiac and intrapulmonary shunt with normal sinus baseline rhythm. There were no atrial premature contractions (APCs), supraventricular tachycardia (SVT), or ventricular tachycardia (VT). The fetal anomaly scan showed a single live intrauterine fetus of 19 weeks of gestation with normal targeted imaging for fetal anomalies.

Based on clinical history, physical examinations, laboratory investigations, and imaging, the diagnosis of basilar artery occlusion with acute infarction of the right pons and right cerebellar peduncle was made, and she was started on Tab.aspirin 150 mg per oral once a day until the delivery, Tab.escitalopram 10 mg per oral once at night, Tab.calcium supplement 500 mg per oral once a day, cremaffin syrup 15 ml per oral at night was administered as per her requirement to relieve constipation and iron and folate supplements with regular obstetric consultation. The patient responded well to the treatment and was able to move in the wheelchair. The patient and her guardians were counseled regarding the risks and benefits of continuing pregnancy, and the patient advocated for continuing it and delivery by vaginal route. The baby was delivered by the vaginal route thereafter, and both the baby and mother faced no complications and are doing well currently.

## Discussion

Cerebral pontine infarctions are uncommon but should be considered in pregnant and non-pregnant women presenting with CNS symptoms indicative of a cerebral vascular accident. The clinical presentation in the general population varies with the infarct’s extent and location. Mild features encompass vertigo, ataxia, dysphagia, dizziness, facial palsy, ophthalmoplegia, aphonia, horizontal gaze paresis, hearing loss, and dysarthria. Severe cases may involve motor hemiparesis of the face, and upper and lower limb brachio-crural ataxia^1,2^. In this case, our patient presents with symptoms like headache, vomiting, altered consciousness, and impaired vocalization followed by sudden weakness in the left upper limb progressing to the left lower limbs. Based on the clinical history and physical examination, viral meningoencephalitis and right-side cerebrovascular accidents were considered differential diagnoses. The diagnosis of right-side cerebrovascular accidents was made, supported by laboratory findings such as increased total leukocytes, neutrophilia, and low hemoglobin, along with imaging studies revealing acute infarction in the right cerebellar hemisphere, pons, and right cerebral peduncle. The combination of clinical history, physical examination, MRI showing basilar artery occlusion, and laboratory parameters contributed to the diagnosis.

The suggested association between pregnancy-related hormones and an elevated risk of strokes in pregnant women has encountered skepticism in the face of subsequent research. Initial hypotheses proposing that heightened levels of vascular endothelial growth factor and basic fibroblast growth factor, induced by hormonal changes, could lead to increased risks have been challenged by recent studies that did not reveal progesterone and estrogen receptors in cases of cavernous malformations. It is interesting to note that there are different possible explanations for strokes during pregnancy, but none of them has been fully proven yet. What we do know is that strokes are not very common during pregnancy – only about 10% of them happen before delivery. Most of them (40%) happen during delivery, while the rest (50%) take place after delivery. The thing is, strokes during pregnancy can be even more complicated because of various factors, such as preeclampsia, eclampsia, and other conditions that can affect both the mother and the baby. It is a complex situation that requires careful management and prevention. In our case, the patient had symptoms during the second trimester, and even though she did not have any history of chronic diseases, she did have a BMI of 24 kg/m^2^, which could be a potential risk factor^7–9^.

As shown in Table 1, initial laboratory findings for our patient with pontine hemorrhage revealed abnormalities in various biological parameters. Notably, there was an elevated total leukocyte count (13 500/cm) with neutrophilia (81%) and lymphocytosis (13%). Hematological markers showed low hemoglobin concentration (11.5 gm%), decreased packed cell volume (32.7%), and reduced RBC count (3.89 million/mm^3^). The patient presented with low levels of sodium (hyponatremia) at 128 mEq/l and potassium (hypokalemia) at 3.5 mEq/l, as well as low serum creatinine at 0.4 mg/dl. Tests for dengue fever were negative, and the levels of antiphospholipid antibodies (anticardiolipin IgG and IgM, β-2 glycoprotein 1 IgG) were within normal ranges. However, fibrinogen levels were elevated at 533 mg/dl. Subsequent days of treatment showed fluctuations in these parameters, highlighting the dynamic nature of the patient’s biological profile during care. In a study conducted by Fan *et al*., 225 patients with primary brain stem hemorrhage (PBSH) were examined retrospectively. The findings suggest that elevated platelet-to-lymphocyte ratio, neutrophil-to-lymphocyte ratio, and admission blood glucose levels were independently associated with unfavorable 90-day functional outcomes of PBSH. The study identified critical thresholds of 59.3 for the platelet-to-lymphocyte ratio, 6.65 for the neutrophil-to-lymphocyte ratio, and 7.81 mmol/l for the admission blood glucose level^10^.

**Table 1:** Laboratory data of significant biological parameters of the patient with pontine hemorrhage on subsequent days of treatment

Biological parameters	Day 3	Day 4	Day 5	Day 6	Day 7	Day 8	Day 9	Day 10	Day11
Total leukocyte count (/mm)	13 500	13 700	10 400	8700	9100	9800	9900	10 400	9700
Neutrophil count (%)	81	73	70	70	67	69	71	68	70.4
Lymphocyte count (%)	13	18	25	24	23	22	20	24	19.0
Hemoglobin (gm%)	11.5	11.2	10.2	11.3	12.4	13.1	12.1	12.4	11.9
Packed cell volume (%)	32.7	32.2	30.2	33.7	35.3	36.6	33.4	34.9	34
RBC count (million/mm^3^)	3.89	3.88	3.66	4.11	4.26	4.42	4.03	4.20	3.97
MCHC (%)	35.17	34.78	33.77	33.53	35.13	35.79	36.23	35.53	35.0
Sodium (mEq/l)	142	147.0	138	138	136	136	138	136	136
Potassium (mEq/l)	3.3	4.0	3.8	3.9	3.9	4.0	3.7	4.0	3.9

Diagnosing hemorrhagic strokes in pregnant women involves clinical assessment and low fetal exposure radiographic imaging, such as CT and MRI. Hemorrhages, including subarachnoid hemorrhage (SAH), may result from ruptured aneurysms and typically present with severe headaches. Diagnostic approaches include uninfused head CT and lumbar puncture for xanthochromia evaluation when CT results are inconclusive. The patient underwent CT and MRI brain imaging to confirm the diagnosis of pontine hemorrhage. The American College of Obstetricians and Gynecologists (ACOG) recommends the use of CT and MRI, with or without contrast, for diagnostic purposes. The use of contrast is recommended when it is likely to provide clear diagnostic benefits. The ACOG guidelines emphasize that there is no proven harm associated with contrast use in CT, and data on gadolinium are inconclusive, with the suggestion to use contrast if it is likely to offer significant diagnostic advantages^11^.

Acute pontine hemorrhage with a significant bleed carries a high risk of fatality and often results in poor outcomes. Managing a particular medical condition during pregnancy requires a team approach involving several specialists including obstetricians, neurologists, neonatologists, neurosurgeons, anesthesiologists, and critical care providers. Due to the sensitive nature of medical interventions during pregnancy, a thorough evaluation of the potential risks and benefits is crucial before administering any care. While the use of rtPA is a treatment option, its application during pregnancy requires meticulous consideration of risks and benefits. Mechanical thrombectomy and embolectomy, commonly employed in non-pregnant women, lack reported instances of use in pregnant patients. Studies have evaluated the safety and efficacy of drugs such as aspirin/extended-release dipyridamole and clopidogrel in the treatment of pontine ischemic infarctions during pregnancy. The results of these studies indicate that antiplatelet therapy, including clopidogrel, is unlikely to increase the risk of congenital anomalies based on animal studies. In the case mentioned, the patient was administered only antiplatelet drugs and showed a positive response to the treatment^12,13^.

Pontine hemorrhage accounts for approximately 10% of all cases of intracerebral hemorrhage (ICH), with outcomes closely tied to the recovery of consciousness, motor function, and respiratory drive, as the cranial nerves V to VIII originate from the pons. Mortality rates range from 30% to 50%, underscoring the severe prognosis associated with this condition. Despite these high mortality rates, conservative management remains the predominant approach for most patients with pontine hemorrhage. In cases where hemorrhage originates in the posterior fossa, guidelines from the American Stroke Association recommend surgical removal of cerebellar hematomas but advise against surgical evacuation of brainstem hematomas due to their association with poor clinical outcomes. Therefore, the treatment and prognostication of brainstem hemorrhage patients pose significant challenges in clinical practice^14^.

If fetal monitoring indicates reassurance, an immediate delivery is not advised. In such situations, when maternal resuscitation and recovery are ongoing, the decision to delay delivery may be considered. It is important to note that the vaginal route of delivery is the preferred method unless obstetrical indications suggest otherwise. In our case, the patient underwent induced vaginal delivery at term, resulting in a live and healthy baby at the time of birth^11^.

## Conclusion

This case of a 33-year-old primipara with a pontine hemorrhage at 15 weeks gestation highlights the challenges of diagnosing and managing rare cerebrovascular events during pregnancy. The patient’s symptoms and subsequent multidisciplinary evaluations underscore the need for prompt and comprehensive care. Despite the rarity of such strokes during pregnancy, early recognition and appropriate treatment, such as antiplatelet therapy, are crucial for ensuring the safety of both mother and fetus and ensuring the normal vaginal mode of delivery. This case underscores the importance of vigilance and preparedness in maternal-fetal medicine. Identifying other differentials and risk factors/etiology in pregnancy is of utmost importance.

## Ethical approval

Since this is a case report no ethical approval is required.

## Consent

Written informed consent is taken and is available for editor on demand.

## Source of funding

Not applicable.

## Author contribution

J.Y., K.B., and I.T.: wrote the original manuscript, and reviewed and edited the original manuscript; K.B., S.S.J., R.K., and A.G.: reviewed and edited the original manuscript.

## Conflicts of interest disclosure

The authors declare no conflict of interest.

## Research registration unique identifying number (UIN)

Not applicable.

## Guarantor

Jeshika Yadav.

## Data availability statement

All relevant data are within the paper.

## Provenance and peer review

Not commissioned, externally peer-reviewed.

## References

[1] Wildman JK Rimawi BH. Cerebral pontine infarctions during pregnancy – a case report and review of the literature. Case Rep Womens Health 2019;21:e00097. 30733924 10.1016/j.crwh.2019.e00097 PMC6358547

[2] James AH Bushnell CD Jamison MG. Incidence and risk factors for stroke in pregnancy and the puerperium. Obstet Gynecol 2005;106:509–516. 16135580 10.1097/01.AOG.0000172428.78411.b0

[3] Meeks JR Bambhroliya AB Alex KM. Association of primary intracerebral hemorrhage with pregnancy and the postpartum period. JAMA Netw Open 2020;3:e202769. 32286658 10.1001/jamanetworkopen.2020.2769 PMC7156993

[4] Nabil EA Badr E Hicham Z. Pontine-bulbar Hemorrhage in a Complicated form of Preeclampsia with Eclampsia and HELLP Syndrome “Case Report.”. Asian J Case Reports Med Heal 2023;1:42–46.

[5] Riley DS Barber MS Kienle GS. CARE guidelines for case reports: explanation and elaboration document. J Clin Epidemiol 2017;89:218–235. 28529185 10.1016/j.jclinepi.2017.04.026

[6] Radhakrishnan M Nagaraja S. Modified Kuppuswamy socioeconomic scale 2023: stratification and updates. Int J Community Med Public Health 2023;10:4415–4418.

[7] Xu YL Liu JT Song YJ. Pregnancy combined with epilepsy and cerebral cavernous malformation. Chin Med J (Engl) 2017;130:619–620. 28229997 10.4103/0366-6999.200533 PMC5339939

[8] Dias MS Sekhar LN. Intracranial hemorrhage from aneurysms and arteriovenous malformations during pregnancy and the puerperium. Neurosurgery 1990;27:855–65; discussion 865–6. 2274125 10.1097/00006123-199012000-00001

[9] Yamada S Nakase H Nakagawa I. Cavernous malformations in pregnancy. Neurol Med Chir (Tokyo) 2013;53:555–560. 23979052 10.2176/nmc.53.555

[10] Chen D Tang Y Nie H. Primary brainstem hemorrhage: a review of prognostic factors and surgical management. Front Neurol 2021;12:727962. 34566872 10.3389/fneur.2021.727962 PMC8460873

[11] Committee Opinion No. 723: Guidelines for Diagnostic Imaging During Pregnancy and Lactation. Obstet Gynecol 2017;130:e210–6. 10.1097/AOG.0000000000002355 28937575

[12] De Santis M De Luca C Mappa I. Clopidogrel treatment during pregnancy: a case report and a review of literature. Intern Med 2011;50:1769–1773. 21841343 10.2169/internalmedicine.50.5294

[13] Smith WS Sung G Saver J. Mechanical thrombectomy for acute ischemic stroke: final results of the Multi MERCI trial. Stroke 2008;39:1205–1212. 18309168 10.1161/STROKEAHA.107.497115

[14] Kim M Cho S You SH. A prognostic model of pontine hemorrhage based on hemorrhage volume and location. J Neurointensive Care 2021;4:21–29.

